# Application of the Length of Index and Ring Fingers to Estimate the Stature

**DOI:** 10.7759/cureus.66497

**Published:** 2024-08-09

**Authors:** Venkatesan M, Hima Shaheed, Sneha S, Sheikh Thufyl

**Affiliations:** 1 Department of Forensic Medicine and Toxicology, Sri Ramachandra Institute of Higher Education and Research, Chennai, IND

**Keywords:** regression model, anthropology, index finger length, ring finger length, stature

## Abstract

Introduction:Stature contributes as a crucial element of an individual's physical appearance and can be instrumental in establishing their identity. In cases where the body is extensively mutilated, decomposed, or reduced to skeletal remains, stature becomes an essential component in identifying the unknown by means of measuring the skeletal remains. Its estimation relies on the principle that an individual’s height has a definite and linear relationship with specific body parts and long bones. This process, together with assessing age, sex, and race constitutes the essential components of the anthropological protocol. Stature estimation can be accomplished through both anatomical and mathematical approaches. The present study clearly defines regression models for height estimation from finger lengths. The formula derived can prove particularly valuable in Medico-legal scenarios, as it can be applied effectively even when only a portion of the body is accessible.

Aim: The purpose of the present study is to estimate the stature of individuals by measuring the length of the index and ring fingers.

Materials and method: The current study acquired three measurements, such as stature, right/left index finger length (RIFL/LIFL), and ring finger length (RFL), from 220 samples, including 110 males and 110 females, respectively, between the age groups of 20 and 60 years.

Result: The application of the length of the index and ring finger in forensic investigations holds significance due to their potential as reliable predictors of an individual’s height. According to the findings of the study, males showed significantly higher stature than females. A statistically significant correlation was also observed (p-value = 0) between stature and finger lengths (IFL, RFL) in both hands. The highest correlation coefficients were found for the left RFL (r = 0.688) in females and the LIFL (r = 0.552) in males. Additionally, males showed significantly longer index and RFL than females. Linear regression models for the estimation of stature from ring and index finger length were also derived successfully.

Conclusion:* *The results obtained from the present study exhibit potential use to evaluate the utility of measuring index and RFLs for determining stature and predicting the precision of regression models by employing those parameters. The models derived from this study can serve as corroborative evidence for identifying mutilated body parts or unknown remains.

## Introduction

Forensic anthropology is a sub-field of physical anthropology that applies scientific tools and techniques for the identification and analysis of human remains in medico-legal investigations. The branch helps deal with the estimation of race, sex, age, and stature making it possible for forensic anthropologists who gather information from skeletal remains to solve crimes at a faster rate [1,2].

Stature estimation is one of the essential biological parameters in the identification of unidentified body parts. Stature refers to a person’s height, particularly the measurement used to describe an individual’s vertical dimension from the base of the feet to the top of the head [3,4]. The link between skeleton dimensions and living height allows for the calculation of stature. Most studies reveal a definite and linear association between a person's height and numerous bodily components or long bones, with reasonable accuracy.

Stature is commonly determined from the skeleton in one of the following two ways: By measuring all bones composing the components of height, aggregating those measurements, and compensating for the missing soft tissue, or by employing a regression formula using the measurements of a whole bone. Other methods include employing incomplete limb bones, non-limb bones, and alternative statistical methods [5,6].

Another intriguing avenue for height estimation lies in the examination of hand dimensions, particularly the lengths of the index and ring fingers [7,8]. The finger length ratio method has been explored as a non-invasive alternative to estimate height, drawing on the idea that finger proportions may exhibit correlations with overall body size. Estimating the stature of human remains assists in the identification process by allowing forensic anthropologists to compare the estimated height with available missing people’s records or information provided by families, thus narrowing down potential matches and aiding in the identification of the deceased [9,10].

The objective of the current study is to measure the lengths of index and ring fingers in volunteers who have given their consent and correlate the acquired data with the subjects' stature. Consequently, create equations for linear regression to determine height.

## Materials and methods

Sample area

The cross-sectional study was conducted at Sri Ramachandra Institute of Higher Education and Research (SRIHER), Chennai, India.

Sample size and period of the study

A total of 220 samples were considered for the study, including 110 males and 110 females, respectively, for two months from June 2023 to August 2023.

Inclusion criteria

Physically fit individuals willing to give consent between the age group of 20 and 60 years were included. The age group was chosen based on previous literature where it has been stated that males reach average adult height by the age of 18-20 years and females by the age of 16-18 years, while height loss accelerates in the age group above 60 years.

Exclusion criteria

The exclusion criteria included any deformity of fingers or spine leading to alterations in height.

Study methodology

After obtaining ethical clearance from the Sri Ramachandra Institute of Higher Education and Research Institutional Ethical Committee, the participants were selected for the study based on inclusion and exclusion criteria. Consent was obtained from the subjects after a thorough explanation of the study. Three measurements were taken including stature, index finger length (IFL), and ring finger length (RFL) from each hand, respectively.

Stature

**Figure 1:**
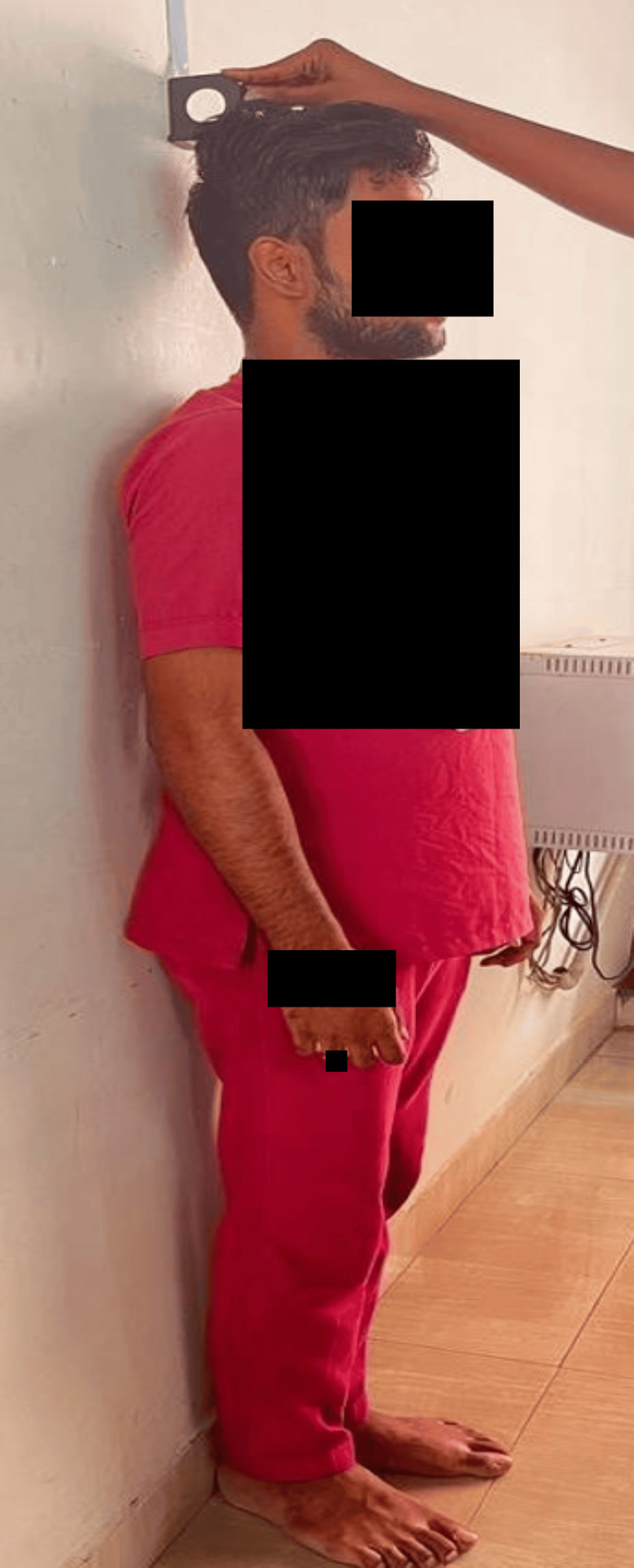
Measuring the height of an individual using a stadiometer

The subject was first made to stand in an erect posture against a wall, in which the stadiometer was priorly fixed and the vertical measurement was taken without any wear on the foot or accessories on the head. The feet axis was placed parallel or slightly divergent and the head was held in the Frankfort horizontal plane while measuring using the stadiometer. The sliding horizontal piece was adjusted accordingly to measure the height of the individuals. It was taken into care that no pressure was exerted over the sliding horizontal piece since this was a contact measurement (Figure 1).

**Figure 2:**
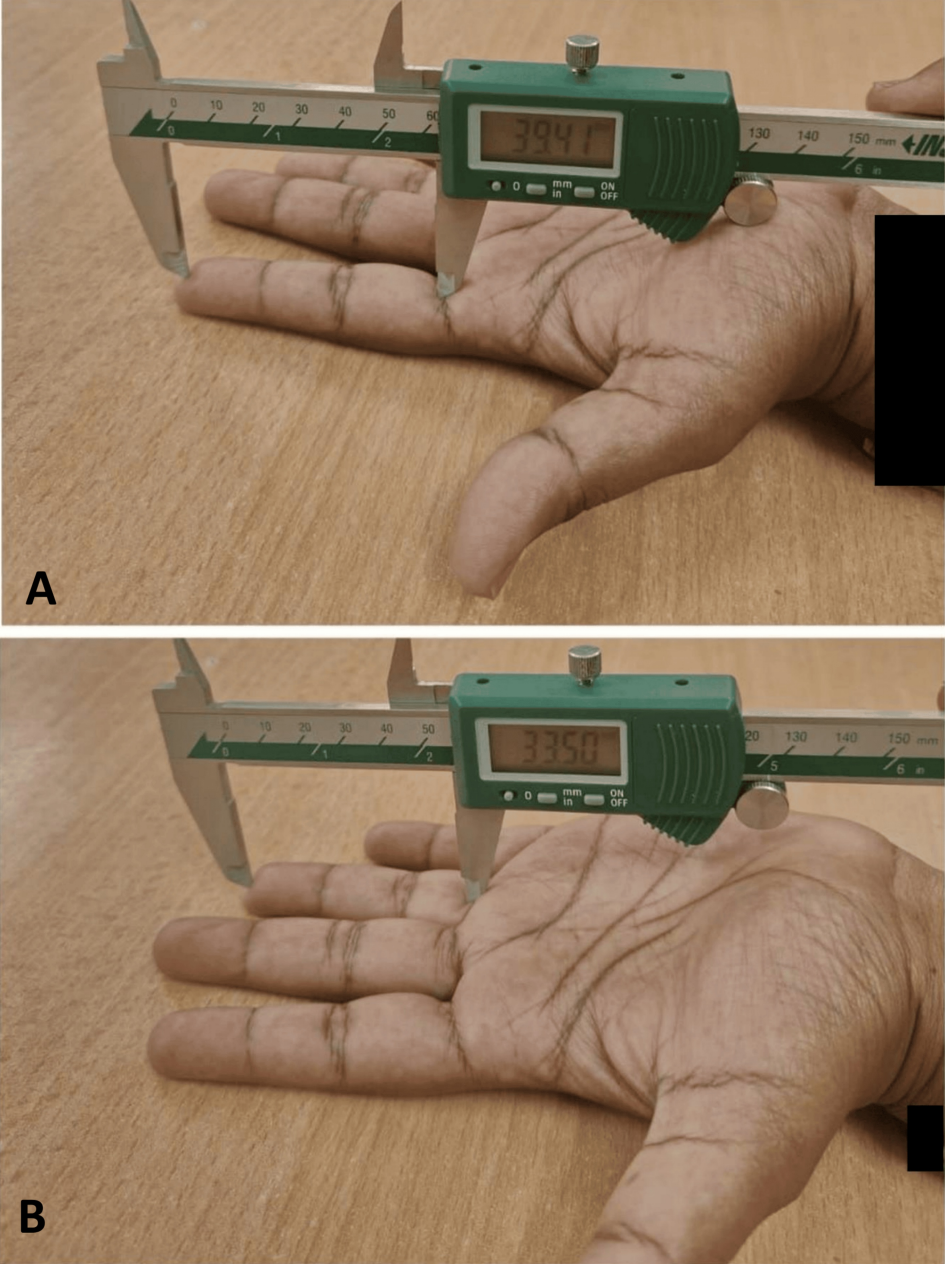
Measuring index and ring finger length using a digital vernier caliper A) index finger length; B) ring finger length

After the height had been measured, the subject was made to sit in a comfortable position to obtain finger measurements. Two finger lengths namely IFL and RFL were taken from each hand, respectively (Figure 2).

Finger lengths

First, the right hand of the subject was placed on a flat surface (to avoid any folding) by stretching their fingers wide. Measurement of IFL was taken between mid-points of the proximal crease at the base of the index finger to the tip of the index finger while RFL was taken between the mid-point of the proximal crease at the base of the ring finger to the tip of the ring finger using digital sliding caliper and the same procedure was followed with the left hand, respectively. All the measurements were obtained in centimeters and were noted down in the proforma for further examination.

Statistical analysis

The collected data was analyzed statistically to determine the correlation between stature and finger lengths using SPSS Statistics for Windows, Version 17 (Released 2008; SPSS Inc., Chicago, United States). Various variables such as stature, IFL, and RFL were correlated using Pearson’s correlation coefficient following which linear regression models were derived for stature estimation using the acquired data. The mean and standard deviation for estimation of stature from index and RFL in the right and left hand were also calculated.

## Results

This cross-sectional study was conducted in the Department of Forensic Medicine and Toxicology, SRIHER in which the length of index and ring fingers of all the volunteers were collected and the values were tabulated for analysis.

Table 1 represents the mean values and standard deviation values for each of the variables used for the study. Here, the dependent variable was considered to be the stature while the independent variables considered were RRF, RIF, LRF, and LIF. The stature obtained ranged between 159.5 cm to 193.5 cm and 137.5 cm and 175.8 cm in males and females, respectively. The mean height of males and females was 174.68 cm and 157.71 cm, respectively. The means of RRF, RIF, LRF, and LIF were 7.47, 7.27, 7.46, and 7.32, respectively, in males while 6.79, 6.64, 6.77, and 6.68, respectively, in females. RFL was found to be larger than IFL in both males and females. Finger length measurements (IFL, RFL) were significantly larger in males than females in both hands. Standard deviation is a measure of how dispersed the data is in relation to the mean. The standard deviations of RRF, RIF, LRF, and LIF ranged between 0.48 to 0.51 in males and 0.40 to 0.42 in females, respectively. The standard deviation showed higher values in males when compared to females.

**Table 1:** Descriptive statistics of various variables in both the population RRF: right ring finger; RIF: right index finger; LRF: left ring finger; LIF: left index finger

Variables	Sex	Mean	Std. deviation
Age	Male/female	28.16/26.51	21.837/9.122
Height	Male/female	174.688/157.713	6.8188/6.8170
RRF	Male/female	7.471/6.793	0.5080/0.4079
RIF	Male/female	7.2701/6.6482	0.48202/0.41414
LRF	Male/female	7.461/6.779	0.5151/0.4215
LIF	Male/female	7.324/6.681	0.4914/0.4119

Table 2 shows the correlation of different variables with stature in the male population. A statistically significant correlation was observed (p-value = 0) between stature and finger lengths (IFL, RFL) in both hands. Pearson correlation coefficient for RIF, RRF, LIF, and LRF was 0.531, 0.529, 0.552, and 0.523, respectively. The correlation coefficient was found to be higher for IFL than RFL. Among the four variables, the left index finger (LIF) showed a high positive correlation with stature in males.

**Table 2:** Pearson correlation between different variables in the male population RRF: right ring finger; RIF: right index finger; LRF: left ring finger; LIF: left index finger

Variables	Pearson correlation coefficient	Significance (2- tailed)
Height	1	0.000
RRF	0.529^*^	0.000
RIF	0.531^*^	0.000
LRF	0.523^*^	0.000
LIF	0.552^*^	0.000
^*^: correlation is significant at the 0.01 level (2-tailed)		

Table 3 shows the correlation of different variables with stature in the female population. A statistically significant correlation was observed (p-value = 0) between stature and finger lengths (IFL, RFL) in both hands. Pearson correlation coefficient for RIF, RRF, LIF, and LRF was 0.654, 0.687, 0.649, and 0.688, respectively. The correlation coefficient was found to be higher for RFL than IFL. Pearson correlation for stature and finger lengths was higher among females than males. Among the four variables, left ring finger length (LRFL) showed a high positive correlation with stature in females.

**Table 3:** Pearson correlation between different variables in the female population RRF: right ring finger; RIF: right index finger; LRF: left ring finger; LIF: left index finger

Variables	Pearson correlation coefficient	Significance (2- tailed)
Height	1	0.000
RRF	0.687^*^	0.000
RIF	0.654^*^	0.000
LRF	0.688^*^	0.000
LIF	0.649^*^	0.000
^*^: correlation is significant at the 0.01 level (2-tailed)		

The "p" value for all the above-mentioned variables is 0.00 for both sexes which is indicative that all these parameters can be considered for stature estimation with high significance.

The general linear regression equation is y = a + (bx), where, "y" denotes stature, "a" is a constant and "b" is the regression coefficient, and "x" is the variable of different finger lengths in males and females. Table 4 represents the linear regression formula calculated from the study for males and females to estimate the skeletal stature from each parameter.

**Table 4:** Regression equations derived to estimate stature using different variables in males and females "y" denotes stature and "x" is the variable of different finger lengths

Males	
Right ring finger length	y = 121.674 + (7.096x)
Right index finger length	y = 120.077 + (7.512x)
Left ring finger length	y = 123.069 + (6.919x)
Left index finger length	y = 118.575 + (7.662x)
Females	
Right ring finger length	y = 79.744 + (11.478x)
Right index finger length	y = 86.090 + (10.773x)
Left ring finger length	y = 82.293 + (11.125x)
Left index finger length	y = 86.001 + (10.734x)

## Discussion

The present study revealed that males exhibit significantly higher stature than females. Moreover, male adolescents included in the study showed notably larger index fingers and RFLs compared to female counterparts. The study of Kanchan et al. [11] on South Indian children did not find any statistically significant sex differences in index and RFLs obtained between males and females. The observed sex differences in dimensions between males and females have been attributed to the earlier skeletal maturity in girls than boys, leading to boys having around two more years of physical growth. Additionally, cultural factors, such as the differential upbringing of the two sexes, may influence growth and development.

Previous studies conducted on hand dimensions in diverse adult populations also confirm greater hand dimensions in males than females. For instance, Agnihotri et al. [12] in the Mauritian population, Sanli et al. [13] in the Turkish population, and Kanchan et al. [14] in the South Indian population reported larger index and RFLs in males than in females. Notably, another study by Kanchan et al. on South Indian adolescents reported a significantly larger RFL in males, though the index finger length did not show any statistically significant sex differences. The present study establishes a positive correlation between stature with IFL and RFL in the adult population.

Rastogi et al. (2008) [15] reported a positive correlation between stature and middle finger length in an adult population while Jee and Yun (2015) [16] revealed that middle finger length in males and RFL in females had the greatest correlation with height among Korean population, respectively. In the current study, it has been observed that the index finger length had the highest correlation than the RFL in males, while vice-versa in females. Of all the four variables considered, the highest correlation coefficients (r) were found for the left index finger length (LIFL) in males and LRFL in females.

The aim of comparing the degree of correlation between stature and finger length in adolescents with that obtained for other body parts in adult populations from the region is to understand if there are any similarities. The results indicate that the relationship between stature and index and RFL in adolescents is comparable to what has been observed in cases of hand length, foot length, head length, and horizontal circumference of the head in adult Indian populations.

Regression models were derived for stature estimation in the present study. Interestingly, the standard error of estimate for predicted stature was higher in males than females, suggesting that stature estimation is more accurate in females. The findings highlight a statistically significant relationship between stature and finger lengths (IFL, RFL) in adolescents. These preliminary results suggest that living stature can be reasonably predicted from index and RFLs.

In cases where children and adolescents are victims of crime or disasters estimating their age remains crucial for identification. Regression models for stature estimation can be applied once the age of the deceased is established to obtain their biological profile. Given that identifying commingled mutilated remains presents a challenge for forensic experts, further research on stature estimation among adolescents is proposed. This will help to better understand the amount of variation and accuracy of stature estimation formulae among adolescent age groups. Continued research on this subject, especially in different populations worldwide, will provide valuable information for forensic anthropologists in reconstructing stature to aid in forensic investigations.

## Conclusions

The application of the length of the index and ring finger in forensic investigations holds significance due to their potential as reliable predictors of an individual’s height. According to the findings of the present study, it can be noticed that males tend to have longer finger lengths and height when compared to females. It can also be noted that left finger lengths such as LIFL and LRFL showed more correlation to stature in males while right finger lengths such as right index finger length (RIFL) and right ring finger length (RRFL) showed more correlation to stature in females. Linear regression models for the estimation of stature from ring and index finger length were also derived successfully. Thereby, this study can help in the approximate estimation of stature even if single parameters such as RRFL, RIFL, LIFL, and LRFL may be obtained from a scene of the crime and serve as corroborative evidence in cases obtaining mutilated body parts, unknown remains identification, etc. However, the study may have a few limitations which can limit the accuracy of the prediction. Still, such limitations can be sorted by further research on the subject, particularly on different populations and age groups to provide valuable information for forensic experts in stature estimation.
